# Associations between modifiable risk factors and hepatocellular carcinoma: a trans-ancestry Mendelian randomization study

**DOI:** 10.1186/s12885-024-12525-x

**Published:** 2024-07-10

**Authors:** Xiaoxia Wei, Chenglei Yang, Qiuling Lin, Moqin Qiu, Qiuping Wen, Zihan Zhou, Yanji Jiang, Peiqin Chen, Xiumei Liang, Ji Cao, Juan Tang, Yuying Wei, Hongping Yu, Yingchun Liu

**Affiliations:** 1 Department of Clinical Trial Base, Guangxi Medical University Cancer Hospital, Nanning, Guangxi China; 2 Department of Hepatobiliary Surgery, Guangxi Medical University Cancer Hospital, Nanning, Guangxi China; 3 Department of Respiratory Oncology, Guangxi Medical University Cancer Hospital, Nanning, Guangxi China; 4 Department of Experimental Research, Guangxi Medical University Cancer Hospital, Nanning, Guangxi China; 5 Department of Cancer Prevention and Control, Guangxi Medical University Cancer Hospital, Nanning, Guangxi China; 6 Department of Scientific Research, Guangxi Medical University Cancer Hospital, Nanning, Guangxi China; 7 Department of Disease Process Management, Guangxi Medical University Cancer Hospital, Nanning, Guangxi China; 8 Key Laboratory of Early Prevention and Treatment for Regional High Frequency Tumor (Guangxi Medical University), Ministry of Education, Nanning, Guangxi China; 9 Key Cultivated Laboratory of Cancer Molecular Medicine of Guangxi Health Commission, Guangxi Medical University Cancer Hospital, Nanning, Guangxi China

**Keywords:** Hepatocellular carcinoma, Mendelian randomization, Modifiable risk factors, Trans-ancestry

## Abstract

**Background:**

Potentially modifiable risk factors for hepatocellular carcinoma (HCC) have been investigated in observational epidemiology studies in East Asian and European populations, whereas the causal associations of most of these risk factors remain unclear.

**Methods:**

We collected genome-wide association summary statistics of 22 modifiable risk factors in East Asians and 33 risk factors in Europeans. Genetic summary statistics of HCC were sourced from the Biobank Japan study (1,866 cases and 195,745 controls) for East Asians, and the deCODE genetics study (406 cases and 49,302 controls) and the UK Biobank (168 cases and 372 016 controls) for Europeans. Two-sample Mendelian randomization (MR) analyses were performed independently for East Asian and European populations.

**Results:**

In East Asians, genetically predicted alcohol frequency, ever drinkers, aspartate aminotransferase (AST), hypothyroidism, chronic hepatitis B, and chronic hepatitis C, metabolic dysfunction-associated steatotic liver disease (MASLD), and autoimmune hepatitis were significantly associated with an increased HCC risk (*P* < 0.05/22). Among European population, alanine transaminase, AST, MASLD, percent liver fat, and liver iron content were significantly associated with a higher risk of HCC (*P* < 0.05/33). The replication dataset and meta-analysis further confirmed these results.

**Conclusions:**

Although East Asian and European populations have different factors for HCC, their common modifiable risk factors AST and MASLD for HCC, offer valuable insights for targeted intervention strategies to mitigate society burden of HCC.

**Supplementary Information:**

The online version contains supplementary material available at 10.1186/s12885-024-12525-x.

## Introduction

Hepatocellular carcinoma (HCC) is the predominant type of liver cancer, ranking the sixth in terms of incidence and the third in mortality among malignant tumors worldwide [1]. HCC is typically diagnosed at an advanced stage, with a 5-year survival rate of approximately 18% [2], posing a heavy global health burden. The prevalence of HCC differs across geographic regions, with the highest incidence in Asia and Africa, whereas it is relatively rare in Europe and North America. The observed differences in the ethnic and geographical distribution of HCC are mainly attributed to specific risk factors, with hepatitis B virus (HBV) or hepatitis C virus (HCV) predominating in Asia, whereas alcohol and metabolic dysfunction liver disease (MASLD) dominates Europe and North America [3]. The identification of causal risk factors is of great significance for early detection and precise prevention of HCC. In particular, improving the understanding of the causality and effect sizes of different risk factors can optimize strategies for HCC prevention. Therefore, a systematic assessment of causal risk factors of HCC is urgently needed to reduce global health burden of HCC, especially modifiable risk factors.

Previous observational studies have reported many potential modifiable factors for HCC, including lifestyle factors (e.g., cigarette smoking, alcohol drinking) [4, 5], serum biomarkers (e.g., C-reactive protein, serum iron) [6, 7], and liver and metabolism-related factors (e.g., viral hepatitis, MASLD) [8, 9]. Nevertheless, certain modifiable factors, such as cigarette smoking [10] and alcohol drinking [11, 12], are inconsistently associated with risk of HCC. Moreover, the causality for these associations is yet to be definitively established, as much of the existing evidence is derived from observational studies that are susceptible to potential residual confounding and reverse causation [13]. In recent years, Mendelian randomization (MR) analysis has emerged as a robust tool for causal inference in epidemiological studies [14]. MR utilizes genetic variants as instrumental variables (IVs) to ascertain the causal relationships between exposures and outcome phenotypes [15]. Because the random assortment of genetic alleles during conception, MR serves as a methodological analog to randomized clinical trials, thus minimizing risks of confounding and reverse causation [16]. Although MR in investigating the causal relationships between modifiable risk factors has been applied to many cancers, very few to HCC. Herein, we performed an MR analysis to examine associations between potentially modifiable risk factors and HCC risk in East Asian and European populations, providing additional insights into the etiology of HCC and the development of preventive strategies.

## Methods

### Study design

**Fig. 1:**
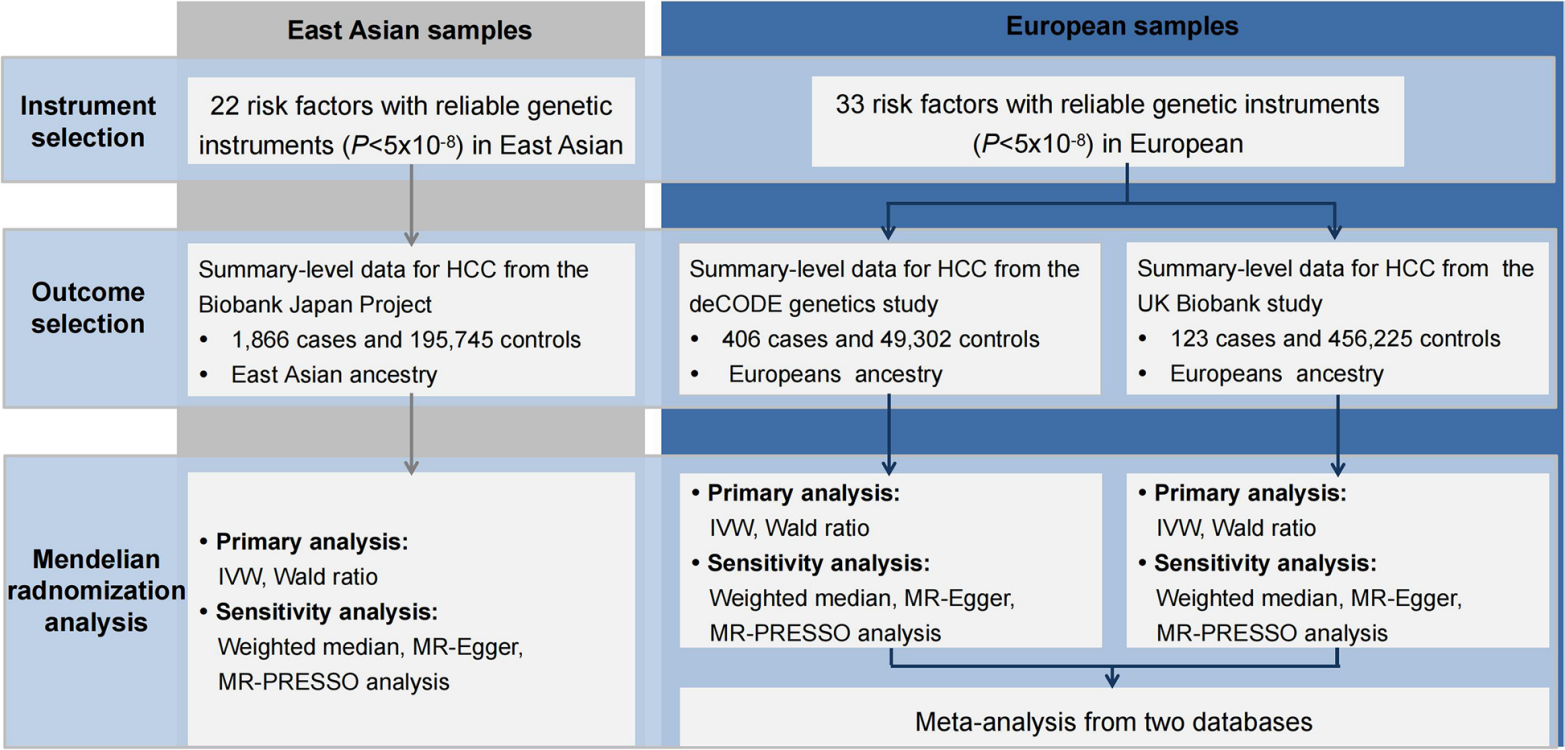
Overview of the study design

An overview of the study design is detailed in Fig. 1. First, a total of 22 modifiable risk factors in East Asians and 33 risk factors in European populations were used, and IVs for each modifiable risk factor were obtained from published genome-wide association studies (GWASs). Second, genetic summary statistics of HCC were obtained from the Biobank Japan (BBJ) study for East Asian ancestry, and the deCODE genetics study and the UK Biobank (UKB) study for European ancestry. Third, we performed MR analyses to assess the causal relationships between genetically proxied modifiable risk factors and HCC risk independently for East Asian and European populations. In Europeans, initial analyses were performed using the deCODE genetics study dataset, followed by replication analyses using the UKB dataset, and both datasets were subsequently used for a meta-analysis to increase the statistical power. All original GWASs included in the current study were approved by the relevant institutional review boards.

### Exposure Data

After reviewing the literature about the epidemiology of HCC, we included the previously reported modifiable risk factors of HCC for the current study, including lifestyle factors (smoking, drinking, physical activity, sleep habits, and education level), serum parameters (serum liver enzymes, C-reactive protein, serum iron, and lipid traits) and metabolic comorbidities (obesity traits, Type 2 diabetes mellitus [T2DM] and related trait, thyroid diseases and related trait, and liver diseases and related trait). By searching the publicly available GWAS summary statistics, we extracted genetic variants associated with 22 modifiable risk factors from East Asian ancestry studies and 33 risk factors from European ancestry studies. Details about the GWASs of modifiable risk factors are provided in Supplementary Tables 1–2.

### Genetic instrument selection

The analysis was restricted to SNPs associated with each trait at the GWAS significance level (*P* ≤ 5 × 10^−8^) in East Asian and European populations. Subsequently, we performed SNP clumping analysis based on linkage disequilibrium (LD) with an *r*^2^ threshold of > 0.001 within 10 Mb to eliminate SNPs in high LD. Given that small amounts of missing data have a limited impact on the results, a few missing instrumental variables were not replaced by proxy variants. To minimize the potential bias stemming from weak IVs, we calculated the *F* statistic (*F* = beta^2^/se^2^) for each SNP to measure the strength of the IVs. SNPs with an *F* statistic exceeding 10 were considered strong instruments [17], while those with an *F* statistic ≤ 10 were excluded from the MR analysis. Power calculations were conducted using an online tool to evaluate the ability to detect an odds ratio (OR) of 0.80 or 1.20 with a Type I error rate of 0.05% (http://cnsgenomics.com/shiny/mRnd/) [18, 19]. The power of each modifiable risk factor on HCC is detailed in Supplementary Tables 1–2, and the genetic instruments of these traits are provided in Supplementary Tables 3-4.

### Outcome data

In East Asians, genetic association of HCC was obtained from the GWAS comprising 197,611 participants (1,866 cases and 195,745 controls) in BioBank Japan [20]. Diagnosis of HCC was conducted by attending physicians at each collaborating institution. In Europeans, the summary-level data on HCC was obtained from the deCODE genetics study (406 cases and 49,302 controls) [21] and UK Biobank (168 cases and 372 016 controls) [22]. HCC was defined by international classification diseases-10 (ICD-10) code C22.0. Detailed information about the outcome is described in Supplementary Table 5.

### Statistical Analysis

MR analyses were performed in East Asians and Europeans, separately. The core assumptions for the MR analysis are illustrated in Supplementary Fig. 1, as described in detail elsewhere [23, 24]. In the primary analysis, the inverse variance weighted (IVW) method was used, and the Wald ratio was used as the final assessment of causal association, when only one SNP was available [25]. The weighted median [26], MR-egger regression [27], and Mendelian randomization pleiotropy residual sum and outlier (MR-PRESSO) [28] methods were used as sensitivity analyses to assess the robustness of the findings. Heterogeneity across estimates of SNPs for each risk factor was assessed by Cochran's Q value [29]. The *P*-value for the MR-Egger intercept was used to assess the horizontal pleiotropy [30]. A meta-analysis was performed to combine estimates from two European data sources. If significant heterogeneity (*P* < 0.05) was observed, a fixed effects model was used to summarize the instrumental variable estimates for each exposure, otherwise a random effects model was used. Results were reported as odds ratios (ORs) with 95% confidence intervals (CIs) to indicate the HCC risk associated with a one-unit increase in exposures. To account for multiple testing, a conservative Bonferroni-corrected *P* value of 2.27 × 10^−3^ (0.05/22, as 22 risk factors were assessed) for East Asians and 1.52 × 10^−3^ (0.05/33, as 33 risk factors were assessed) for Europeans was considered statistically significant, with a nominal *P* value < 0.05 and a Bonferroni-corrected *P*-value ≥ 0.05 being considered a suggestive association. *P* value < 0.05 was defned as the signifcance level for replication among Europeans. All statistical analyses were performed using the TwoSampleMR [31], and MR-PRESSO [28] packages in the R statistical software version 4.0.2.

## Results

### Modifiable risk factors and HCC in the East Asian population

**Fig. 2:**
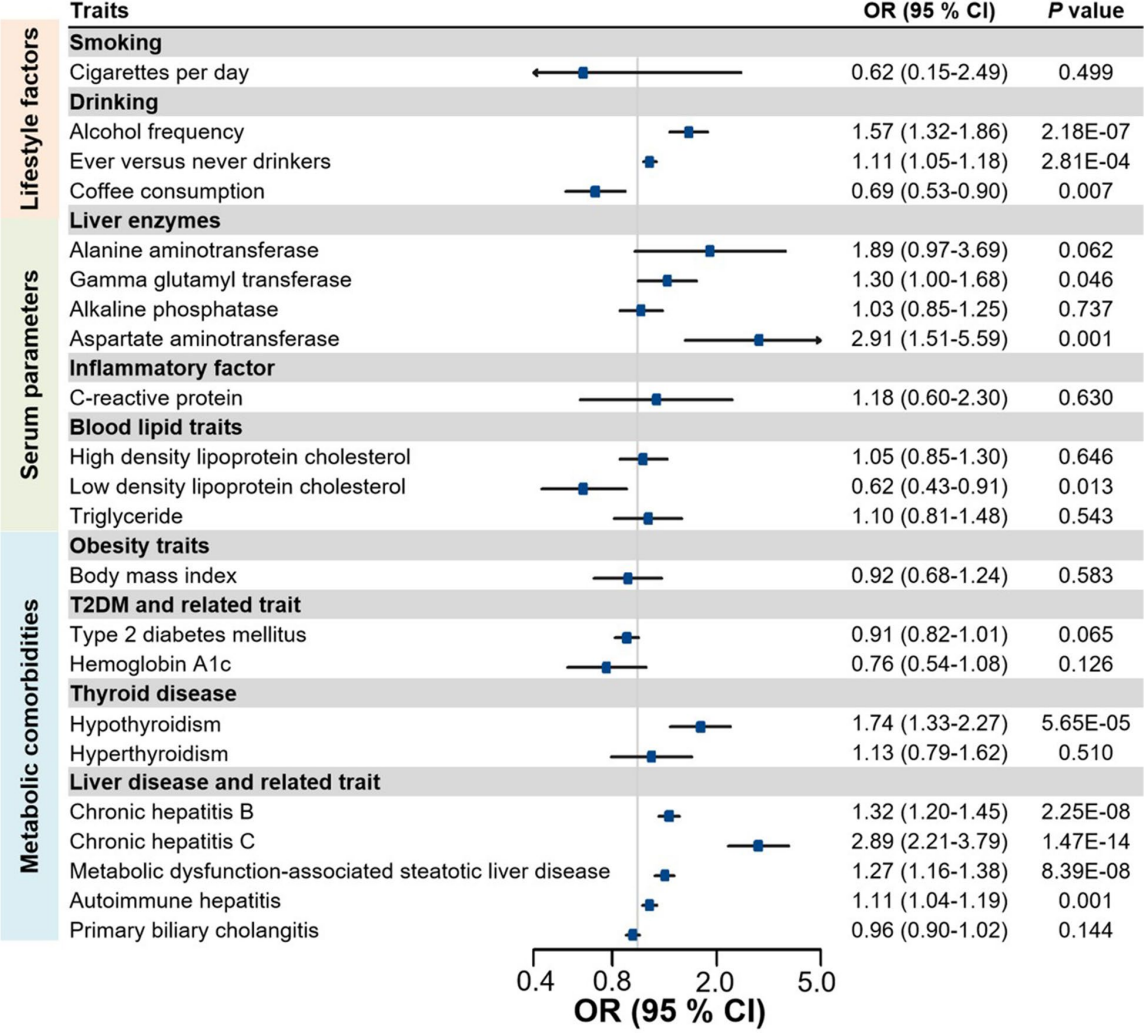
Association of genetic liability to modifiable risk factors with risk of HCC in East Asian individuals in the Biobank Japan. CI, confidence interval; OR, odds ratio

The associations between genetically predicted modifiable risk factors and HCC risk in the East Asian population are illustrated in Fig. 2. Regarding the lifestyle factors investigated, genetically predicted alcohol frequency was positively associated with risk of HCC (OR = 1.57, 95% CI: 1.32–1.86). Similarly, ever drinkers had a higher HCC risk, compared with never drinkers (OR = 1.11; 95% CI: 1.05–1.18). We also noted a suggestive association between a higher coffee consumption and a decreased risk of HCC (OR = 0.69; 95% CI: 0.53–0.90). In our restricted analysis of serum parameters, per 1‐SD increase of aspartate aminotransferase (AST) levels was significantly associated with an increased risk of HCC (OR = 2.91, 95% CI: 1.51–5.59). Gamma glutamyl-transferase levels were suggestively associated with an elevated risk of HCC for a 1-SD increase (OR = 1.30, 95% CI: 1.01–1.68), while low density lipoprotein cholesterol (LDL-cholesterol) levels were suggestively associated with a decreased risk of HCC (OR = 0.62, 95% CI: 0.43–0.91). An increased risk for HCC was associated with genetically predicted metabolic comorbidities, including hypothyroidism (OR = 1.74, 95% CI: 1.33–2.27), chronic hepatitis B (CHB) (OR = 1.32; 95% CI: 1.20–1.45), chronic hepatitis C (CHC) (OR = 2.89, 95% CI: 2.21–3.79), MASLD (OR = 1.27, 95% CI: 1.16–1.38), and autoimmune hepatitis (OR = 1.11, 95% CI: 1.04–1.19), but not for other risk factors. The findings remained robust and consistent in the sensitivity analyses (Supplementary Table 6). Although Cochran’s Q tests indicated heterogeneity in the causal estimates for some modifiable risk factors, the MR-Egger intercept test did not reveal any signs of horizontal pleiotropy (Supplementary Table 7).

### Modifiable risk factors and HCC in the European population

**Fig. 3:**
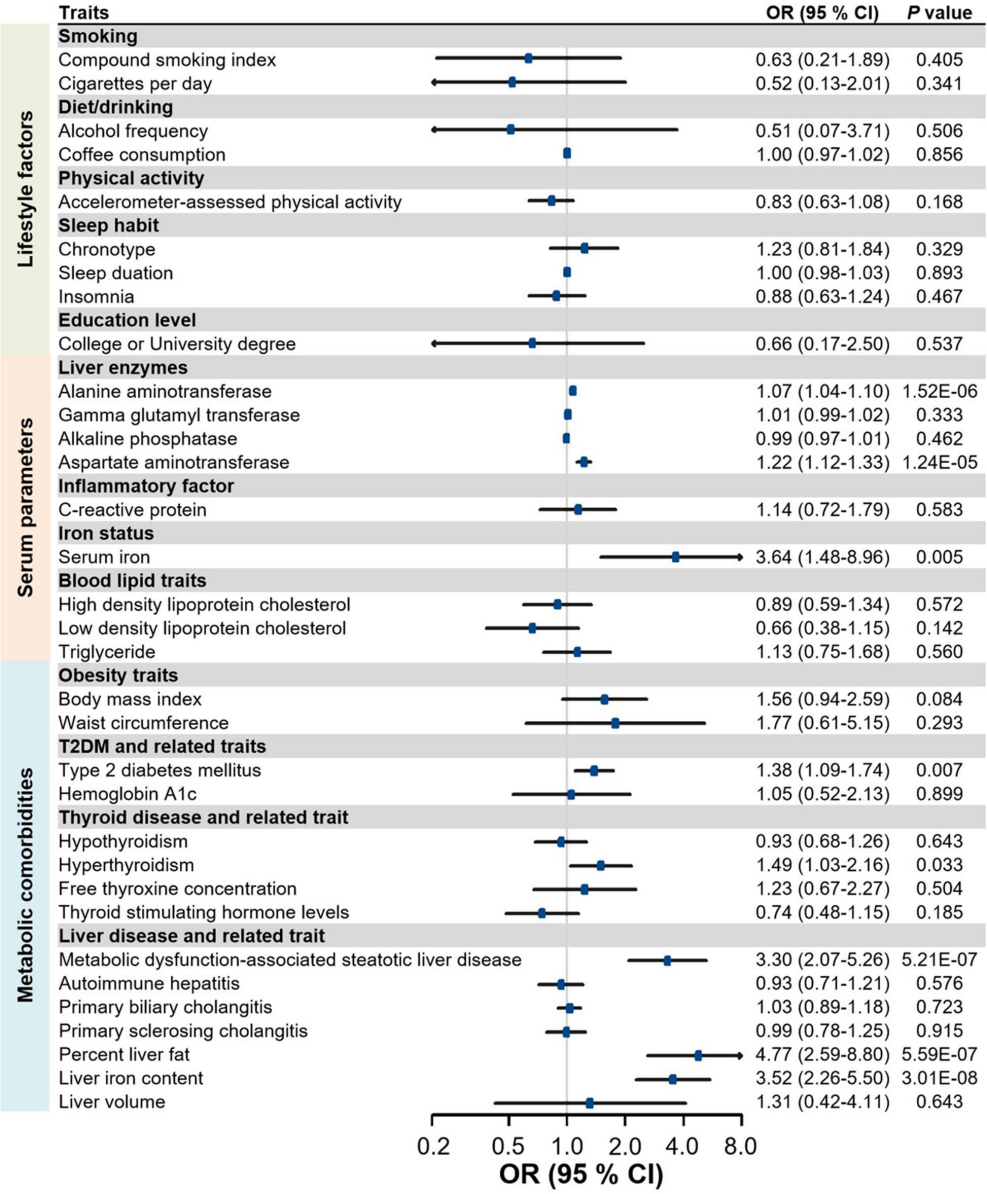
Association of genetic liability to modifiable risk factors with risk of HCC in European individuals in the deCODE genetics study. CI, confidence interval; OR, odds ratio

In participants of European ancestry, our analyses revealed no evidence of significant associations between lifestyle factors and HCC risk (all *P* > 0.05; Fig. 3). Regarding serum parameters, a 1-SD increase in alanine transaminase (ALT) was associated with a 1.07-fold increased risk of HCC (OR = 1.07, 95% CI: 1.04–1.10); for AST levels, the OR of HCC increased by1.22 fold (95% CI: 1.12–1.33). Additionally, there was a suggestive evidence indicating that genetically predicted serum iron levels were associated with an increased risk of HCC for a 1-SD increase (OR = 3.64, 95% CI: 1.48–8.96). Furthermore, an increased risk of HCC was associated with metabolic comorbidities, including MASLD (OR = 3.30, 95% CI: 2.07–5.26), percent liver fat (OR = 4.77, 95% CI: 2.59–8.80), and liver iron content (OR = 3.52, 95% CI: 2.26–5.50). We also observed suggestive associations with increased HCC risk for T2DM (OR = 1.38, 95% CI: 1.09–1.74) and hyperthyroidism (OR = 1.49, 95% CI: 1.03–2.16). Sensitivity analyses yielded similar causal associations (Supplementary Table 8). Heterogeneity was observed in the MR analyses of LDL-cholesterol, with no evidence of horizontal pleiotropy observed for any of the modifiable risk factors (SupplementaryTable 9).

The causal associations in the replication datasets are presented in Supplementary Fig. 2 and **S**upplementary Table 10. The association with HCC was replicated in UKB dataset for ALT (OR = 1.10, 95% CI: 1.02–1.18), AST (OR = 1.30, 95% CI: 1.09–1.55), MASLD (OR = 15.66, 95% CI: 6.81–36.02), percent liver fat (OR = 25.50, 95% CI: 10.79–60.28), and liver iron content (OR = 6.17, 95% CI: 2.08–18.31). The heterogeneity was observed in the MR analyses for AST, serum iron, LDL-cholesterol, triglyceride, T2DM and primary sclerosing cholangitis, but possible pleiotropy was detected in the replication analysis for high density lipoprotein cholesterol and LDL-cholesterol (Supplementary Table 11).To increase the power of the analysis, we performed a meta-analysis for the deCODE genetics study and UKB datasets. The summarized results of the meta-analysis are displayed in Supplementary Fig. 3. The results further confirmed that risk of HCC was positively associated with ALT (OR = 1.07, 95% CI: 1.05–1.10), AST (OR = 1.24, 95% CI: 1.14–1.34), MASLD (OR = 6.91, 95% CI: 1.50–31.72), percent liver fat (OR = 10.72, 95% CI: 2.08–55.35), and liver iron content (OR = 3.82, 95% CI: 2.53–5.77). However, the heterogeneity between results of the two studies was found in the analyses of cigarettes per day, free thyroxine concentration, MASLD, and percent liver fat (*P*_het_ < 0.05).

## Discussion

To the best of our knowledge, this is the first large MR study to systematically investigate the causal associations between modifiable risk factors and the risk of HCC in both East Asian and European populations. Utilizing large-scale GWAS summary statistics within a unified MR framework, we have identified distinct sets of modifiable risk factors for HCC. In this trans-ethnic MR study, we found that higher genetically predicted AST and MASLD were associated with increased HCC risk in the two ethnic populations. We also provide some evidence supporting causal associations of alcohol frequency, ever drinkers, hypothyroidism, CHB, and CHC, and autoimmune hepatitis with the risk of HCC in the East Asian population, as well as ALT, percent liver fat, and liver iron content with the risk of HCC in the European population.

The causal patterns of risk factors revealed consistent effects of AST and MASLD on HCC in both East Asian and European populations. Specifically, we found that elevated levels of AST were associated with an increased risk of HCC, consistent with reported finding that a decrease of AST may be negatively associated with the risk of HCC [32]. Similarly, a Taiwanese study has identified that elevated AST and ALT levels were statistically significant independent predictors of HCC risk [33]; and another European study reported that elevated AST and ALT levels were positively associated with HCC risk [34]. Furthermore, our study showed that ALT was a risk factor for HCC in the European population. Although liver enzymes are functional markers of liver tissue damage, our study showed that elevated liver enzymes were also a risk factor for HCC development, suggesting an etiological connection between liver damage and HCC susceptibility.

Recently, increasing evidence suggests that MASLD is a risk factor for HCC [35]. The current study provided compelling evidence for a causal association between MASLD and HCC in East Asian and European populations, which is consist with a recent MR study [36]. We also observed a direct causal association between the percentage of liver fat measured by magnetic resonance imaging (MRI) and HCC risk, further reinforcing this finding. The biological mechanisms underlying MASLD leading to HCC involve the release of inflammatory cytokines (such as TNF-α, IL6) and reduced adiponectin levels that promote insulin resistance, which in turn inhibits fatty acid oxidation, leading to DNA damage and mutations. Additionally, insulin resistance triggers the release of insulin-like growth factor 1 and insulin receptor substrate 1, influencing cell proliferation and apoptosis, contributing to hepatocarcinogenesis [37]. The burden of HCC due to viral hepatitis is decreasing with advances in HBV and HCV treatment, but the prevalence of MASLD-related HCC is rapidly increasing [38]. This trend emphasizes need for public health measures targeting the prevention and management of MASLD.

In East Asian populations, we observed an association between alcohol consumption with HCC risk, consistent with a recent MR study [39]. Viral hepatitis is well-established as a significant factor in HCC, and many previous studies have reported that CHB and CHC are associated with an increased risk of HCC [8, 40]. We have confirmed these findings using MR analyses. This robust evidence solidifies hepatitis infection as a causal risk factor for HCC, emphasizing the critical importance of early detection and prompt treatment of chronic hepatitis infection as a key strategy to mitigate HCC risk in East Asian populations. Multiple studies have reported that abnormal thyroid function plays an important role in the etiology of many liver diseases, including MASLD [41] and fibrosis [42]. In our study, we observed a positive association between genetically predicted hypothyroidism and the risk of HCC. Furthermore, the association between autoimmune hepatitis and a higher risk of HCC has been observed in a previous observational study [43], and this finding was confirmed in our MR study. In European populations, serum iron has been reported in association with HCC risk in previous studies [7], but limited MR studies to date have evaluated the role of serum iron in HCC risk. We provided evidence supporting a suggestive association between higher serum iron levels and an increased HCC risk, this conclusion was further confirmed by MRI measurements of liver iron content in our study. A plausible explanation is that hepatic iron overload may contribute to oxidative stress-induced carcinogenesis processes [44].

There were several strengths in the present study. First, this is the first large study that has relatively comprehensively assessed large numbers of known and suspected risk factors and biomarkers for HCC risk. Second, a main strength of this study is the MR design with data from different populations, which diminishes confounding and reverse causality as well as may mitigate biases arising from population structure. Third, we performed a series of sensitivity analyses to ensure the robustness and reliability of our findings. Nevertheless, the present study has some limitations. First, although we carefully reviewed and summarized factors associated with HCC risk and GWAS-identified SNPs, the limitations in the design of GWASs we used, and the lack of SNPs in some GWASs, may have led to biases in assessing causality. Second, the statistical power was low in some analyses due to the small variance in exposure explained by IVs (e.g., cigarettes per day and compound smoking index), resulting in underpower to detect associations between the exposure and outcome variable. Additional studies are needed to assess these risk factors in the future. Third, there is a partial sample overlap between exposure and outcome variables, which could introduce a weaker instrument bias [45]. Nevertheless, the SNPs we used were selected based on stringent genome-wide significance thresholds, and all estimated *F* statistics exceeded 10, suggesting that the influence of partial sample overlap on our findings is likely minimal. Fourth, HCC diagnosis is defined differently in the two populations (e.g., attending physician in East Asians *vs*. ICD codes in Europeans) may introduce a degree of misclassification bias. Finally, the availability of HCC cases was relatively limited, particularly among European populations, but we had combined estimates from two separate European databases to enhance the statistical power. Future studies with larger samples sizes of HCC cases are warranted.

By systematically assessing the causal evidence for modifiable risk factors on HCC in individuals of East Asian and European ancestry, the current study has demonstrated that there are eight risk factors for HCC in East Asian populations; in the European population, we identified five HCC risk factors. We found that AST and MASLD were common risk factors in both populations. The current study provides valuable insights for future research and public health interventions.

### Supplementary Information

Supplementary Material 1.

Supplementary Material 2.

## Data Availability

Only publicly available data were used in this study, and data sources and handling of these data are described in the Materials and Methods, and online supplemental information. Further information is available from the corresponding author upon request.

## Abbreviations

ALT: Alanine transaminase
AST: Aspartate aminotransferase
BBJ: Biobank Japan
BMI: Body mass index
CHB: Chronic hepatitis B
CHC: Chronic hepatitis C
CIs: Confidence intervals
FDR: False-discovery rate
GWASs: Genome-wide association studies
HCC: Hepatocellular carcinoma
ICD-10: International classification diseases-10
IVs: Instrumental variables
IVW: Inverse variance weighted
LD: Linkage disequilibrium
LDL-cholesterol: Low density lipoprotein cholesterol
MR: Mendelian randomization
MR-PRESSO: Mendelian randomization pleiotropy residual sum and outlier
MASLD: Metabolic dysfunction-associated steatotic liver disease
ORs: Odds ratios
T2DM: Type 2 diabetes mellitus
UKB: UK Biobank
