# Supplementary material for: Associations between modifiable risk factors and hepatocellular carcinoma: a trans-ancestry Mendelian randomization study
Source: BMC Cancer. 2024 Jul 10;24:820. doi: 10.1186/s12885-024-12525-x (PMC11234530; doi:10.1186/s12885-024-12525-x)
Supplement: Supplementary file 1 — Supplementary Material 1. [file 12885_2024_12525_MOESM1_ESM.docx]

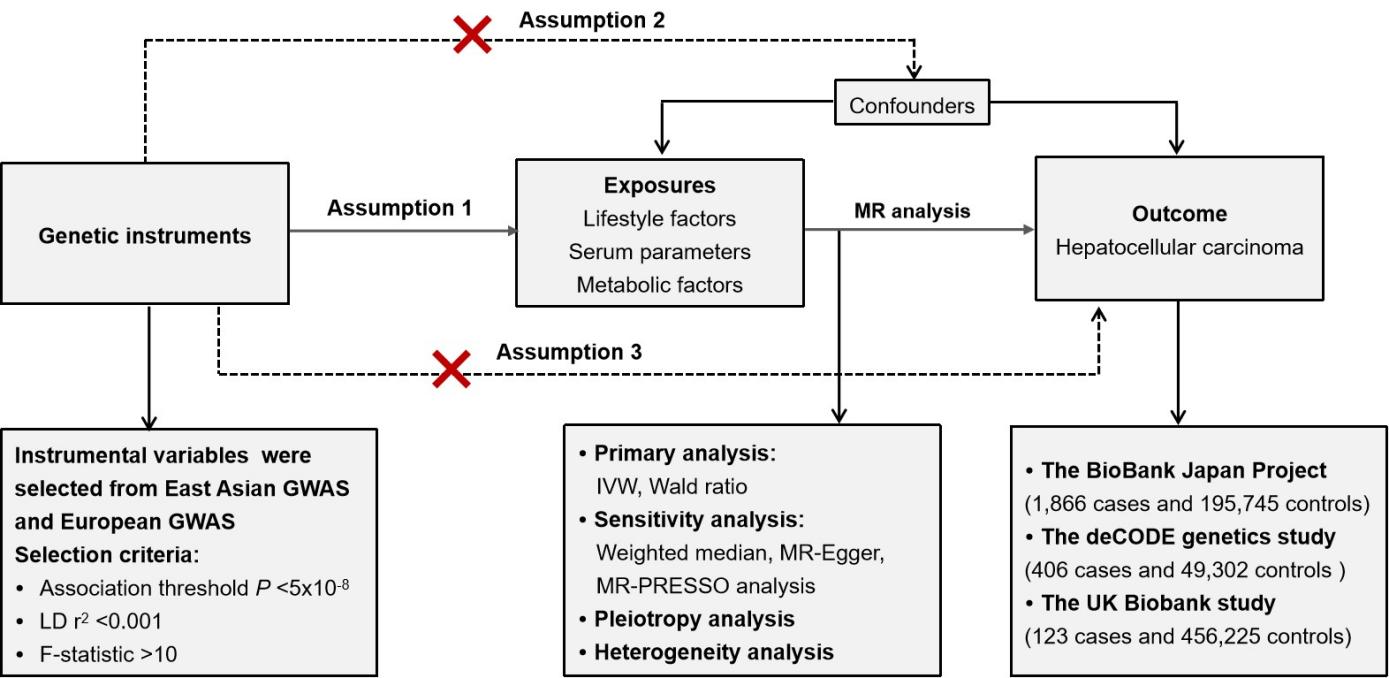


**Supplementary Figure 1.** Strategies to satisfy the three assumptions of IVs in MR analysis. (i) genetic variants should be associated with the risk factor of interest; (ii) genetic variants are not associated with confounding factors; and (iii) genetic variants affect outcomes only through the exposure and not through other pathways.


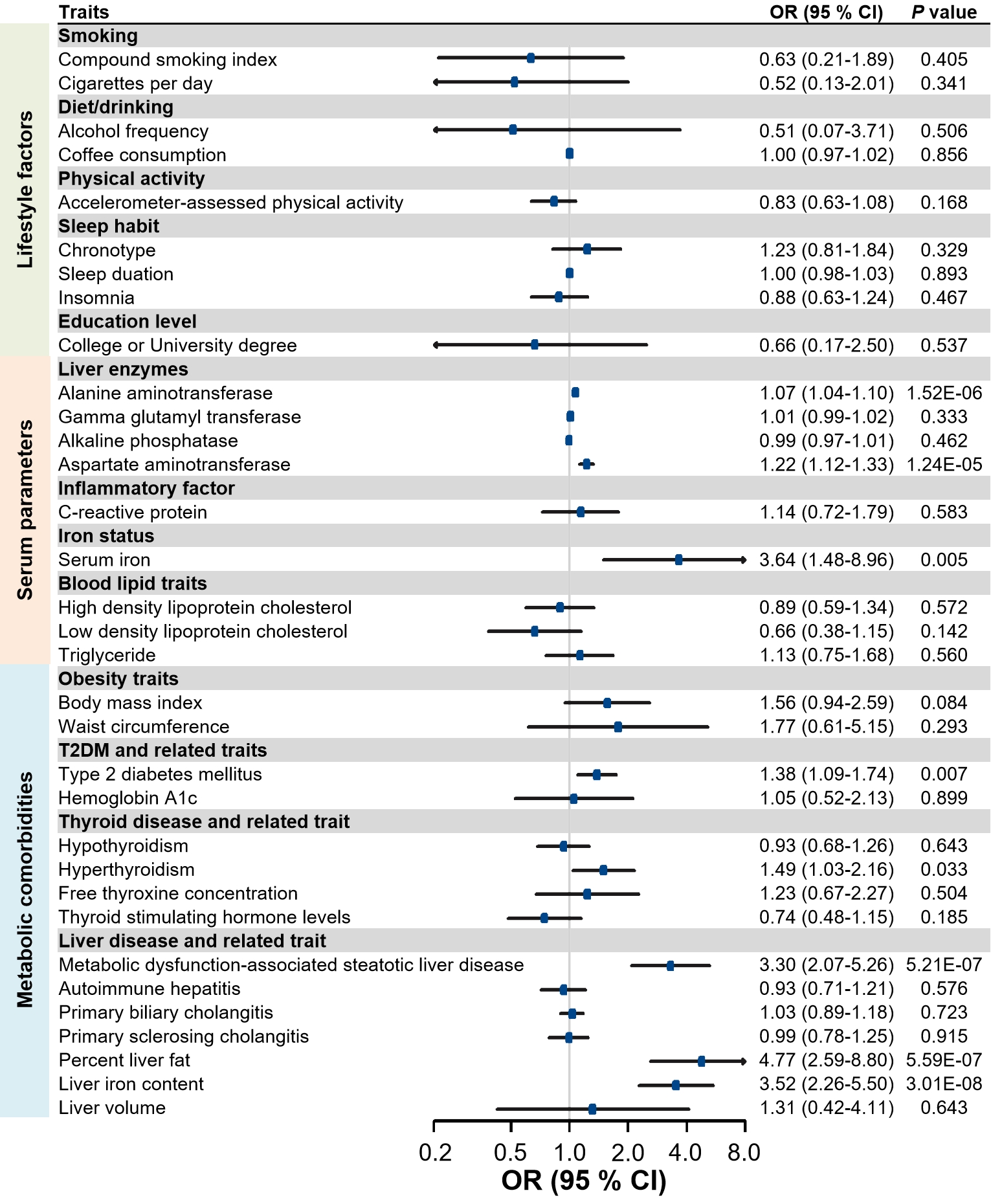


**Supplementary Figure 2.** Association of genetic liability to modifiable risk factors with risk of HCC in European individuals in the UK Biobank study. CI, confidence interval; OR, odds ratio


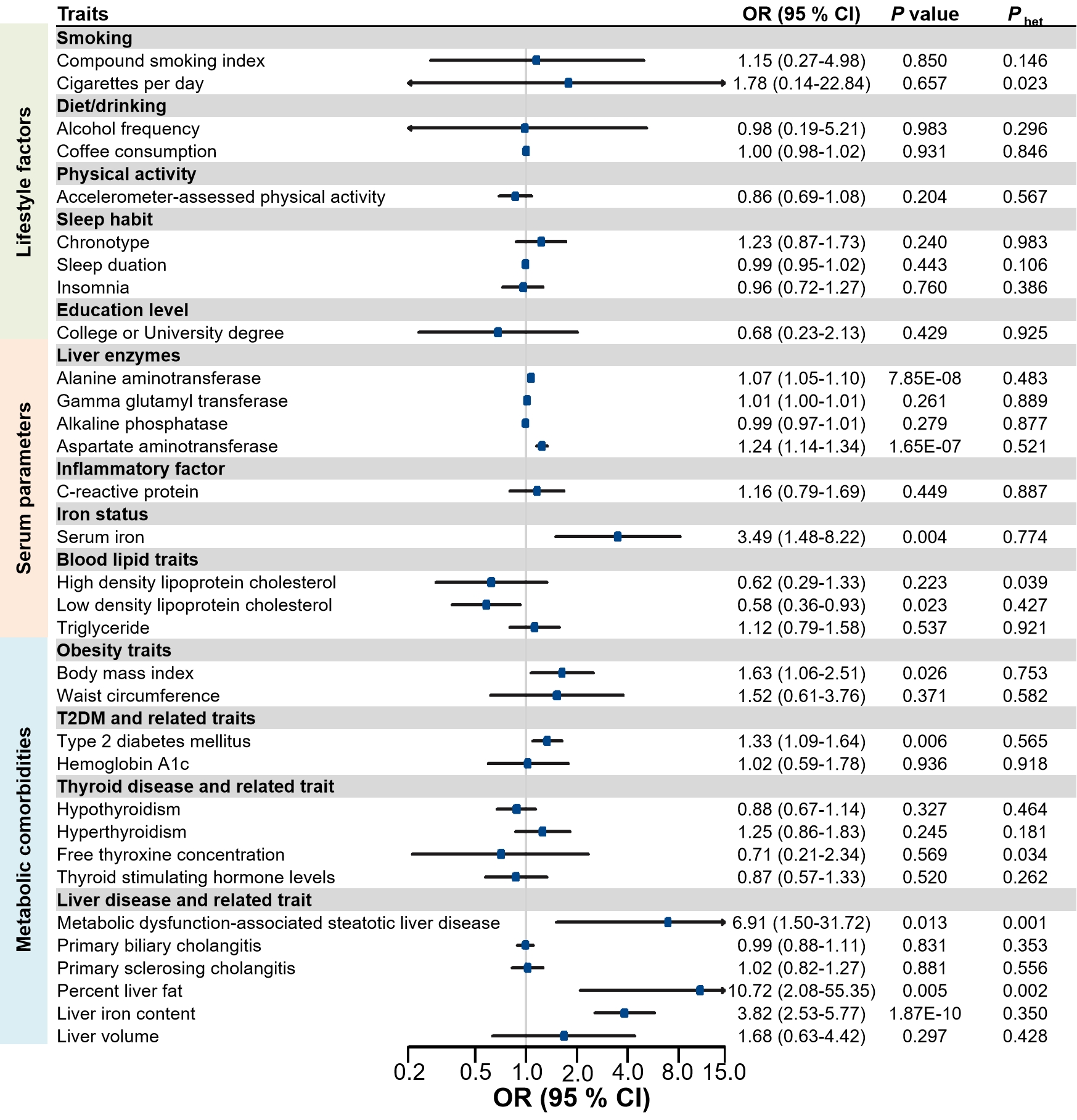


**Supplementary Figure 3.** Association of genetic liability to modifiable risk factors with risk of HCC in European individuals in the meta-analysis of deCODE genetics study and the UK Biobank study. CI, confidence interval; OR, odds ratio; het, heterogeneity
